# Supplementary material for: NEFFy: a versatile tool for computing the number of effective sequences
Source: Bioinformatics. 2025 Jun 3;42(6):btaf222. doi: 10.1093/bioinformatics/btaf222 (PMC13242184; doi:10.1093/bioinformatics/btaf222)
Supplement: btaf222_Supplementary_Data [file btaf222_supplementary_data.zip › NEFFy supplementary file.pdf]

Supplementary File of NEFFy: A Versatile Tool for Computing the Number of Effective Sequences

Maryam Haghani[1,*], Debswapna Bhattacharya[1], and T. M. Murali[1]
[1]Department of Computer Science, Virginia Tech, Blacksburg, VA 24061, United States of America.
[*]Email: haghani@vt.edu

Contents

S1 Introduction 4

S2 Options and Features 4
- S2.1 Integration of Existing Features 4
  - S2.1.1 Sequence Similarity 4
  - S2.1.2 Sequence Weights 4
  - S2.1.3 Query Gaps 4
  - S2.1.4 Gappy Position 6
  - S2.1.5 Alphabet 6
  - S2.1.6 Non-Standard Residues 6
- S2.2 Introduction of New Features 7
  - S2.2.1 NEFF Calculation for Multiple MSAs 7
  - S2.2.2 Per-Residue NEFF (Column-Wise NEFF) 7
  - S2.2.3 NEFF Calculation of Multimeric MSAs 7

S3 Exploring NEFF Calculation Tools 9
- S3.1 RaptorX 9
- S3.2 Conkit 9
- S3.3 DeepMSA 10
- S3.4 Gremlin 10
- S3.5 rMSA 10

S4 Analysis and Results 10
- S4.1 Reliability of NEFFy in Comparison with Other Tools 10
- S4.2 Computational Efficiency of NEFFy 15
- S4.3 Scalability Assessment 15
- S4.4 Case Study on Multi-domain Proteins 20

S5 Alternative NEFF Formulations **20**
S5.1 Clustering-Based NEFF calculation 20
S5.2 Entropy-Based NEFF Calculation 21

Table S1: Summary of Supplementary Excel Files and Sheets: This table provides an overview of each supplementary Excel file, detailing the sheet names, descriptions, and corresponding sections in the paper.

**File Name**	**Sheet Name**	**Description**	**Notes**
Supplementary File S1.xlsx	CASP15 NEFF	NEFF values generated by running `NEFFy` and other tools on CASP15 dataset MSAs.	Related to S4.1 (first and second paragraphs).
Supplementary File S1.xlsx	`rMSA` NEFF	NEFF values generated by running `NEFFy` and `rMSA` on `rMSA` dataset MSAs.	Related to S4.1 (third paragraph).
Supplementary File S2.xlsx	Tool Timing (Average)	Average execution time for each tool based on 5 runs on CASP15 data.	Related to S4.2.
Supplementary File S2.xlsx	Scalability (Average)	Average execution time for each tool based on 5 runs across 36 CASP15 MSAs with varying depths.	Related to S4.3.
Supplementary File S3.xlsx	Multi-Domain Analysis	NEFF values for MSAs of entire proteins and individual domains in multi-domain CASP15 targets.	Related to S4.4.

S1 Introduction

NEFFy is a versatile and efficient tool for calculating the number of effective sequences (NEFF) across multiple MSA formats for various types of biological sequences.

A comprehensive user guide, including installation instructions and usage examples, can be found in the documentation at https://maryam-haghani.github.io/NEFFy.

S2 Options and Features

NEFFy is designed to seamlessly incorporate all NEFF calculation options provided by existing tools, as outlined in the subsections below.

S2.1 Integration of Existing Features

S2.1.1 Sequence Similarity

When determining the cutoff for sequence similarity, two approaches can be used: **1.** symmetric and **2.** asymmetric. In the symmetric approach, the similarity cutoff is uniform across all sequences in the MSA and is calculated as the product of sequence length and the given similarity threshold. Sequences are considered similar if the number of position-wise matches between them exceeds this cutoff. In contrast, the asymmetric approach only considers the non-gap positions in each sequence when calculating the number of position-wise matches, resulting in a variable similarity cutoff for each sequence. This leads to an asymmetric evaluation of sequence pairs. The default approach in NEFFy is symmetric, but users can switch to the asymmetric approach by setting is_symmetric to false.
While RaptorX exclusively supports the asymmetric method and Conkit, Gremlin, and rMSA support only the symmetric method, only DeepMSA and NEFFy are capable of handling both approaches.

The pseudo code for computing sequence weights is outlined in algorithm 1.

S2.1.2 Sequence Weights

As described in the paper, NEFF can be considered as the normalized sum of weights for all sequences in an MSA. NEFFy offers the option to return the weight values of the sequences in the MSA instead of the final NEFF. This can be achieved by setting only_weights to true.

S2.1.3 Query Gaps

In some MSA files, such as the sto format, gaps may appear in the query sequence, indicating "gaps aligned to insertions". When calculating NEFF, users may prefer to exclude these gap positions from all sequences in the MSA. While most tools strictly adhere to the original MSA file, including any gaps in the query sequence, NEFFy provides flexibility in handling these gaps. It allows users to either filter them out, along with the corresponding positions in the aligned sequences (the default option), or retain them by setting omit_query_gaps to false.

Algorithm 1 Sequence Weight Calculation

procedure COMPUTESEQUENCEWEIGHT($sequences$, $similarity\_threshold$)
  $sequence\_weight \leftarrow []$
  if $symmetric$ **then**
    $sequence\_cutoff \leftarrow length \times similarity\_threshold$
  else
    for each sequence s in sequences **do**
      $non\_gap[s] \leftarrow$ non-gap positions of sequence s, considering value of $non\_standard\_option$
      $non\_gap\_count[s] \leftarrow$ number of non_gap positions
      $sequence\_cutoff[s] \leftarrow non\_gap\_count[s] \times similarity\_threshold$
    end for
  end if
  Initialize $similar\_sequences$ with all 1s (for the sequence, itself)
  for any sequence s in sequences **do**
    if $symmetric$ **then**
      $similar\_sequences[s] \leftarrow$ count of sequences whose position-wise matches with sequence $s \geq sequence\_cutoff$
    else
      $similar\_sequences[s] \leftarrow$ count of sequences whose position-wise matches with non-gap positions of sequence $s \geq sequence\_cutoff[s]$
    end if
  end for
  for any sequence s in sequences **do**
    $sequence\_weight[s] \leftarrow \frac{1}{similar\_sequences[s]}$
  end for
  return $sequence\_weight$
end procedure

It is also worth noting that the `Conkit` tool can handle gaps in the query sequence for the `a3m` format by offering two distinct options: `a3m-inserts` and `a3m`.

S2.1.4 Gappy Position

Introduced by `Gremlin`, gappy positions in a sequence alignment are those where the gap frequency exceeds a specific cutoff, indicating a higher-than-desired occurrence of gaps. Pseudo code is provided in algorithm 2. By filtering out these gappy positions, the NEFF calculation can focus on more informative and conserved regions of the sequences. The default approach in `NEFFy` includes all positions, treating none as gappy regardless of the number of gaps (`gap_cutoff = 1`). However, users can adjust the `gap_cutoff` parameter to any value greater than 0, up to 1, to manage gappy positions based on the specified cutoff.

Algorithm 2 Gappy Positions

```
procedure HandleGappyPositions(sequences, gap_cutoff)
    depth ← number of sequences
    gappy_positions ← []
    for each position p in alignment do
        gap_count[p] ← gaps in position p across sequences
        if gap_count[p] ≥ gap_cutoff × depth then
            append position p to gappy_positions
        end if
    end for
    for each sequence s in sequences do
        remove elements at positions listed in gappy_positions from sequence s
    end for
end procedure
```

S2.1.5 Alphabet

Each biological sequence is encoded using a valid set of characters to represent its composition. In the case of proteins, this set consists of 20 canonical amino acids, with each amino acid being represented by a specific letter. The list includes: 'A', 'C', 'D', 'E', 'F', 'G', 'H', 'I', 'L', 'M', 'N', 'P', 'Q', 'R', 'S', 'T', 'V', 'W', 'Y'. Additionally, six non-standard amino acids, as detailed in section S2.1.6, also are included within the protein alphabet.
For DNA sequences, the alphabet comprises 'A', 'T', 'C', 'G' along with non-standard nucleic acid, 'N'. Similarly, in the case of RNA sequences, the alphabet consists of 'A', 'U', 'C', 'G' along with non-standard nucleic acid 'N'.
The default option in `NEFFy` is 'protein' alphabet.

S2.1.6 Non-Standard Residues

Non-standard residues refer to those that fall outside the typical set of residues, explicitly 'N' for DNAs and RNAs and 'X', 'B', 'J', 'O', 'U', 'Z' for proteins.

When calculating NEFF and determining sequence weights, various strategies can be applied for handling non-standard residues by configuring the `non_standard_option` parameter:

- **`AsStandard`**: Treat them as standard amino acids, following the approach of `DeepMSA`'s symmetric version.
- **`ConsiderGapInCutoff`**: To consider them as gaps only during sequence cutoff determination (the number of matches per sequence), aligning with `DeepMSA`'s asymmetric method.
- **`ConsiderGap`**: Treat them as gaps both in sequence cutoff calculation and when identifying matching positions between sequences, similar to the methods used by `rMSA` and `Gremlin`.

`NEFFy` is the only tool that supports all these options, with the default being `AsStandard`.

S2.2 Introduction of New Features

In addition to incorporating features from existing tools, `NEFFy` brings new capabilities for NEFF calculation, outlined in the subsections below.

S2.2.1 NEFF Calculation for Multiple MSAs

In all versions of NEFF calculation except for the NEFF of multimer MSA, `NEFFy` can accept multiple MSA input files as long as all sequences have the same length. It merges the sequences from these files in the specified order, removing any duplicates, and then computes the NEFF for the combined MSA.

S2.2.2 Per-Residue NEFF (Column-Wise NEFF)

This new feature computes NEFF for each position in the alignment. Per-residue NEFF values for each position in the MSA are calculated by summing the weights of the sequences that have a residue (i.e., non-gap characters) at that specific position. It is used by tools like AlphaFold [Jumper et al., 2021] for more precise per-residue sequence diversity assessment.

S2.2.3 NEFF Calculation of Multimeric MSAs

`NEFFy` can compute NEFF values for multimeric MSAs (i.e., MSAs corresponding to multiple protein chains). Tools such as AlphaFold-Multimer [Evans et al., 2021] generate these multimeric MSAs. We consider two cases:

1. **Homomers:** Assemblies composed of multiple copies (let us say n) of the same chain. In this case, the multimeric MSA is constructed by concatenating n copies of each row from the individual chain's MSA into a single sequence. Figure S1-a shows an example of a homomeric MSA.
2. **Heteromers:** Assemblies comprising two or more distinct chains. Here, the MSA is structured with a paired sequence alignment at the beginning, followed by rows showing separate,

```
            chain A      chain A
Seq1:    M  K  T  L | M  K  T  L
Seq2:    N  K  T  L | N  K  T  L
Seq3:    M  K  V  L | M  K  V  L
Seq4:    M  R  T  - | M  R  T  -
```

(a) **Homomeric MSA (stoichiometry = A2)**

```
             chain A       chain A    chain B
Paired:
Seq1:    M  K  T  L  |  M  K  T  L  | P  A  G
Seq2:    M  K  T  M  |  M  K  T  M  | P  C  G
Seq3:    M  R  T  -  |  M  R  T  -  | P  -  G

Individual MSA for chain A (block-diagonal):
Seq4:    M  K  S  L  |  -  -  -
Seq5:    M  -  S  L  |  -  -  -

Individual MSA for chain B (block-diagonal):
Seq6:    -  -  -  -  |  P  A  -
Seq7:    -  -  -  -  |  R  A  G
```

(b) **Heteromeric MSA (stoichiometry = A2B1)**

Figure S1: **Schematic representation of multimeric MSAs**. In subfigure **(a)**, the MSA depicts a homomeric assembly (stoichiometry `A2`), which the same chain (chain A) is repeated horizontally two times (two copies of chain A). In subfigure **(b)**, the MSA depicts a heteromeric assembly (stoichiometry `A2B1`), showing a paired MSA block showing two chains (A and B) in the same rows, followed by individual MSAs for each chain in a block-diagonal format. Note that, regardless of how many numbers each chain contributes, only a single unpaired MSA block is in the MSA.

unpaired MSAs for each chain arranged in a block-diagonal format. The paired MSA contains sequences that have non-gap residues in aligned positions for at least two different chains, indicating that these sequences represent two or more distinct chains of the complex, which reflect the sequence diversity across different chains and can show coevolutionary information among them. Importantly, our definition is not tied to any specific pairing strategy. We define the paired portion of the MSA as the initial segment where non-gap residues are present in at least two distinct chains, regardless of how the paired MSA was generated. In contrast, the individual MSAs have non-gap residues only in the aligned positions corresponding to their respective chain, with gaps appearing in the aligned positions for the other chains. In some instances, certain chains may not have an individual MSA, but a paired MSA is always present. Figure S1-b illustrates an example of a heteromeric MSA.

It is important to note that the paired MSA portion is not included in the depth of individual MSAs. In other words, for a protein complex consisting of n monomers, where x denotes

the depth of the paired MSA and y_1 through y_n denote the depths of the individual MSAs for each chain, the total MSA depth is computed as $x + y_1 + \cdots + y_n$. For example, in Figure S1-b, the paired MSA contains 3 sequences, and the individual MSAs for chain A and chain B each have 2 sequences, resulting in a total depth of 3 + 2 + 2 = 7.

Users should provide the stoichiometry of the multimer (using the `--stoichiom` option) so that `NEFFy` can determine whether the assembly is a homomer or a heteromer. As described by the Protein Data Bank [RCSB Protein Data Bank], *"Stoichiometry indicates the number of chains participating in the assembly and whether the assembly is a homomer or a heteromer."* **For homomers**, NEFF is calculated based on the MSA of the individual chain repeated horizontally in the multimeric MSA. **For heteromers**, the user must also provide the lengths of each chain in the complex (using the `--chain_length` option). With these two inputs, `NEFFy` distinguishes between the paired MSA and the unpaired MSAs of each chain and computes the NEFF values separately for each.

S3 Exploring NEFF Calculation Tools

In this section, we will conduct a thorough exploration of the tools featured in Table 1 of the main paper which have integrated NEFF calculations into their core functions. We will delve into the primary purposes each of these tools serve and provide an in-depth exploration of the various features they offer for executing NEFF computations.

S3.1 `RaptorX`

`RaptorX`, a well-established protein structure prediction method developed since 2012, specializes in predicting protein tertiary and contact structures, particularly for sequences lacking close homologs in the Protein Data Bank [Källberg et al., 2012]. `RaptorX` includes an integrated Python helper function for NEFF calculation, called **`Meff`**, which is specifically designed for computing asymmetric NEFF. This tool is primarily designed for handling aligned `a2m` and `a3m` formats, without the specification of biological sequence alphabets. The code is available on GitHub at `https://github.com/j3xugit/RaptorX-3DModeling/tree/master/BuildFeatures/Helpers`.

S3.2 `Conkit`

`Conkit` is a Python library designed to facilitate the management and manipulation of residue-residue contact prediction data [Simkovic et al., 2017]. Among its key functionalities, it provides parsers for different MSA formats, allowing conversion between them, as well as the the capability to calculate NEFF and sequence weights, with a performance boost from Cython for time efficiency. The source code is available on GitHub at `https://github.com/rigdenlab/conkit/blob/master/conkit/core/sequencefile.py`, and the documentation can be found here.

`Conkit` does not specify the biological sequence type for NEFF computation. Additionally, when calculating symmetric NEFF, it gives an integer-valued NEFF value without normalization. Furthermore, `Conkit` can handle gaps in query sequences, but this option is available exclusively in

the a3m format, distinguishing it from the a3m-inserts format, which includes gap positions of the query sequence in MSAs.

S3.3 DeepMSA

DeepMSA is an open-source tool designed for constriction of deep and sensitive MSAs [Zhang et al., 2020]. It achieves this by leveraging homologous sequences and alignments derived from a diverse range of whole-genome and metagenome databases, utilizing complementary hidden Markov model algorithms. DeepMSA includes a built-in feature for NEFF computation in C++, which is exclusively available for the aln format. It offers support for both symmetric and asymmetric NEFF calculation for protein alphabets. The source code can be accessed on GitHub at https://github.com/kad-ecoli/MSAParser/blob/master/calNf.cpp.

S3.4 Gremlin

Gremlin is a method for prediction of residue-residue contacts that uses the power of sequence co-evolution and structural context data using a pseudo-likelihood approach. This allows for more precise prediction in protein structures, even when working with a limited set of homologous sequences [Kamisetty et al., 2013]. Gremlin has been implemented in both Python and C++, and within its code, it includes the capability to compute symmetric NEFF.
When computing NEFF, Gremlin treats all non-standard residues as equivalent to gaps. It can support various biological sequences, including proteins, RNAs, and DNAs. Gremlin offers the feature to exclude gappy positions from the MSA and supports fasta and aln MSA formats. The source code is accessible on GitHub at https://github.com/sokrypton/GREMLIN_CPP/blob/master/gremlin_cpp.cpp.

S3.5 rMSA

rMSA is a hierarchical pipeline designed for conducting sensitive searches and precise alignments of RNA homologs for a RNA sequence [Zhang et al., 2023]. rMSA includes a native feature for computing NEFF values specifically customized for RNA sequences. Research in RNA 3D structure prediction and protein-nucleotide structure prediction has employed rMSA to generate MSAs for RNA sequences [Baek et al., 2022, Feng et al., 2022, Pearce et al., 2022]. The source code can be accessed on GitHub at https://github.com/pylelab/rMSA/blob/master/src/fastNf.cpp.

S4 Analysis and Results

S4.1 Reliability of NEFFy in Comparison with Other Tools

To evaluate the reliability and consistency of NEFFy compared to other tools, we calculated the NEFF value for each MSA file across 93 CASP15 targets. MSA files for these targets were generated using default pipeline of AlphaFold v2.3, which leverages three databases—Uniref90, Mgnify, and BFD—resulting in three separate MSA files per target, totaling 279 files in both STO

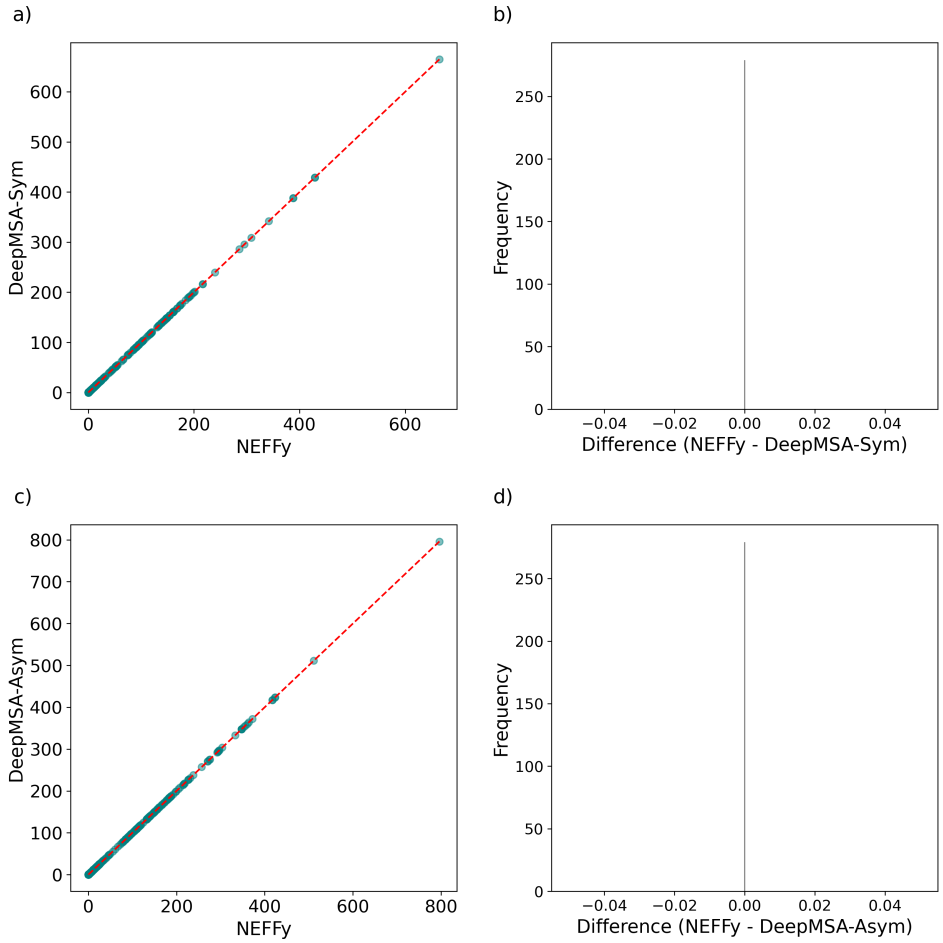

Figure S2: Comparison of NEFF values between `DeepMSA` and `NEFFy` (using parameters corresponding to `DeepMSA`): (**a**, **c**) Scatter plots illustrating NEFF values for symmetric (**a**) and asymmetric (**c**) versions, with the dotted red line representing the identity line (x = y), indicating equal values for both methods. (**b**, **d**) Histograms showing the distribution of differences for symmetric (**b**) and asymmetric (**d**) versions.

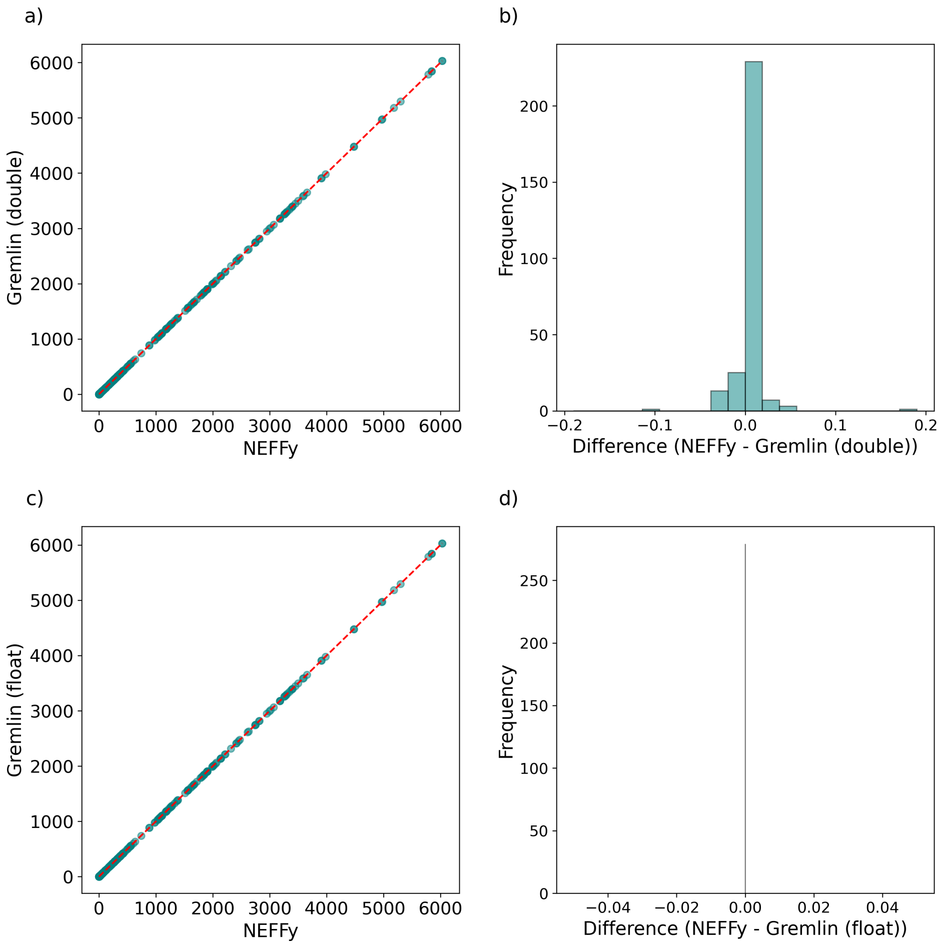

Figure S3: Comparison of NEFF values between `Gremlin`, using `double` (default version) and `float` data types, and `NEFFy` (using parameters corresponding to `Gremlin`) for the CASP15 dataset: (**a**, **c**) Scatter plots illustrating NEFF values for `double` (**a**) and `float` (**c**) configurations, with the dotted red line representing the identity line (x = y), indicating equal values for both methods. (**b**, **d**) Histograms showing the distribution of differences for `double` (**b**) and `float` (**d**) configurations.

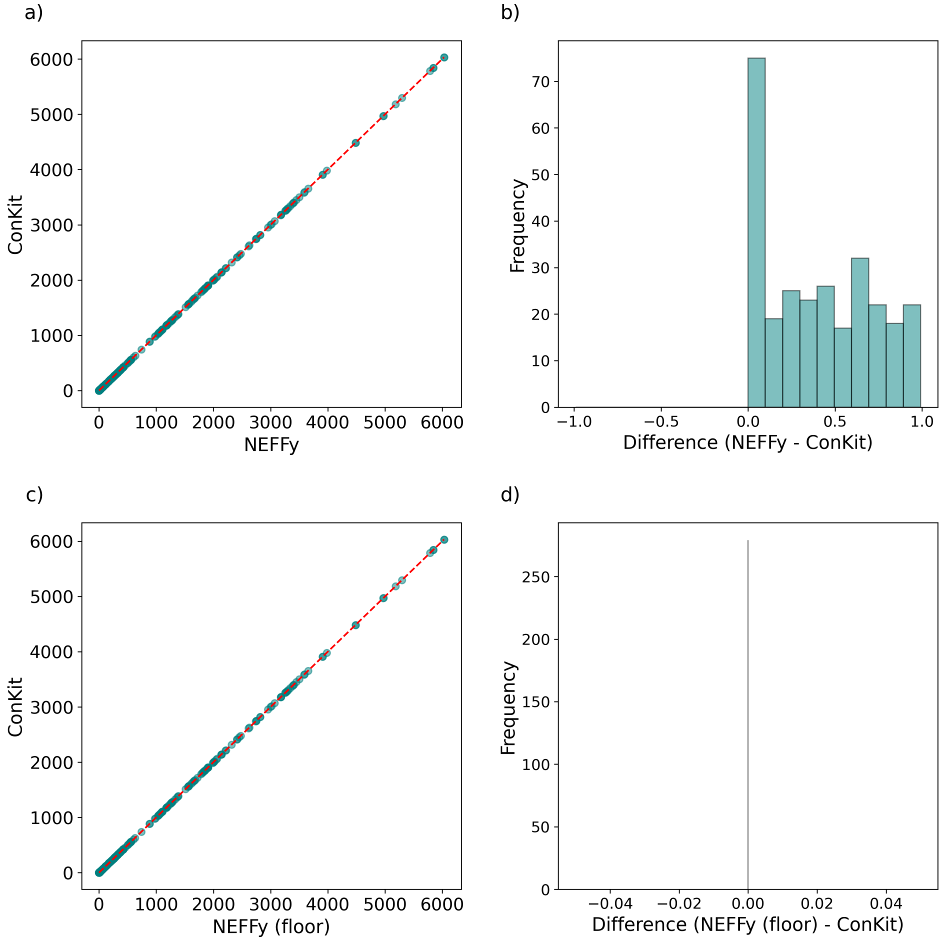

Figure S4: Comparison of NEFF values between `ConKit` and `NEFFy`, evaluated with both default and 'rounded NEFF values' configurations (using parameters aligned with `ConKit`) for the CASP15 dataset: (**a**, **c**) Scatter plots illustrating NEFF values for default (**a**) and 'rounded NEFF values' (**c**) configurations, with the dotted red line representing the identity line (x = y), indicating equal values for both methods.(**b**, **d**) Histograms showing the distribution of differences for default (**b**) and 'rounded NEFF values' (**d**) configurations.

Tool	Mode	Commands
DeepMSA	Symmetric	DeepMSA: **./calNf [file] 0.8 0**<br>NEFFy: **./neff --file=[file]**
	Asymmetric	DeepMSA: **./calNf [file] 0.8 10**<br>NEFFy: **./neff --file=[file] --is_symmetric=false --non_standard_option=1**
Gremlin		Gremlin: **./gremlin_cpp -i [file] -eff_cutoff 0.8 -gap_cutoff 1 -only_neff**<br>NEFFy: **./neff --file=[file] --norm=2 --non_standard_option=2**
Conkit		Conkit:<br>import conkit.io<br>msa = conkit.io.read(file, format)<br>neff = msa.meff<br>depth = msa.nseq<br><br>NEFFy: **./neff --file=[file] --norm=2**
RaptorX		RaptorX: run *RaptorX* python source with threshold = 0.8<br>NEFFy: **./neff --file=[file] --is_symmetric=false --norm=2**
rMSA		rMSA: **./fastNf [file] 0.8 0**<br>NEFFy: **./neff --file=[file] --non_standard_option=2**

Table S2: Commands for calculating NEFF values with each tool and their equivalents when using NEFFy.

and A3M formats. Since some tools do not support NEFF calculation in these formats, we used NEFFy's built-in converter to transform the files into compatible formats for each tool (available at https://doi.org/10.5281/zenodo.14210949). Table S2 provides a summary of the commands required to calculate NEFF values with each tool, including both the commands using the tool and their equivalents when using NEFFy.

Our findings demonstrated complete consistency with both the symmetric and asymmetric options of DeepMSA (Figure S2). For Gremlin, only minor differences were observed due to its use of the double data type, while NEFFy and DeepMSA use float for decimal representation. These differences in data types impact precision and storage size, which can cause slight variations in results during mathematical operations. However, when the data type in Gremlin was changed to float, the results matched entirely (Figure S3). In the case of Conkit, the tool rounds NEFF values to integers, which explains the subtle differences observed in NEFF results. When the NEFF values from NEFFy were rounded, the results aligned with those of Conkit (Figure S4). For RaptorX, we observed slight variations, primarily due to its consistent exclusion of lowercase residues from sequences and its unique method for determining cutoffs by taking the minimum length of each sequence pair during similarity calculations. Additionally, the implementation in Python may introduce minor discrepancies in handling decimal numbers (Figure S5).

Additionally, for rMSA, we used the benchmark dataset described in [Zhang et al., 2023], which includes 361 non-redundant RNA chains collected from the PDB database. We generated MSAs for these chains using rMSA tool (available at https://doi.org/10.5281/zenodo.14210949). We then conducted a comparative analysis by comparing our NEFF values with those generated by the built-in rMSA NEFF computation tool for these files. The NEFFy results showed minor differences compared to the built-in tool, as NEFFy uses the float data type while rMSA uses double. As a test, we changed rMSA's data type to float and obtained identical results (Figure S6).

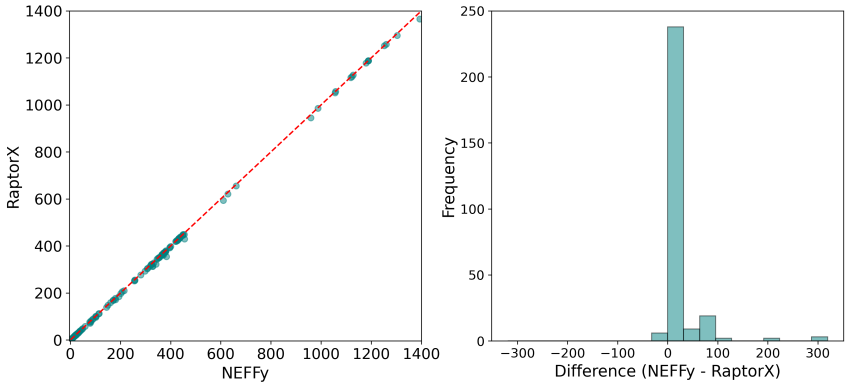

Figure S5: Comparison of NEFF values between `RaptorX` and `NEFFy` (using parameters corresponding to `RaptorX`). (**a**) Scatter plot illustrating NEFF values, with the dotted red line representing the identity line (x = y), indicating equal values for both methods. (**b**) Histogram showing the distribution of differences.

S4.2 Computational Efficiency of `NEFFy`

We used the same set of 279 MSA files generated by AlphaFold for CASP15 targets. Each tool was run on these files five times to record execution times, and the average execution time for each file and tool was calculated. Figure S7 illustrates the distribution of execution times across the various tools.

`RaptorX`, implemented entirely in Python, utilizes nested loops for its similarity calculations. This approach can be inefficient, particularly with large datasets, resulting in significant performance bottlenecks and slower processing speeds. In contrast, `Conkit`, also written in Python, leverages Cython code segments to enhance its performance through C-like syntax and optimizations. Conversely, `DeepMSA`, `Gremlin`, `rMSA`, and `NEFFy` are considerably faster due to their implementation in C++, which allows for compiler optimizations that improve processing speed and overall performance.

Statistical analysis using the one-sided Wilcoxon signed-rank test [Wilcoxon, 1992] further supports these performance differences. `NEFFy` demonstrates comparable performance to `DeepMSA` ($p \approx 0.027$) and `Gremlin` ($p \approx 0.081$), showing no significant difference. However, `NEFFy` significantly outperforms `Conkit` ($p \approx 8.29 \times 10^{-48}$) and `RaptorX` ($p \approx 8.94 \times 10^{-48}$).

S4.3 Scalability Assessment

We used 36 targets from CASP15, each with a UniRef90 MSA of 10,000 sequences, to evaluate NEFF computation for all tools across four depths: 10, 100, 1000, and 10,000. `NEFFy` supports custom depths directly, while for other tools, we used the complete MSA for a depth of 10,000 and extracted the first 10, 100, and 1,000 sequences from the MSA file to calculate NEFF for depths of 10, 100, and 1,000, respectively. We measured the execution time for each tool and depth across five separate runs and calculated the average time for each target from these five executions to evaluate scalability as depth increased.

Fig. S8 demonstrates the scalability of five different methods—`NEFFy`, `DeepMSA`, `Gremlin`, `Conkit`, and `RaptorX`—as a function of increasing MSA depth on a logarithmic scale for both

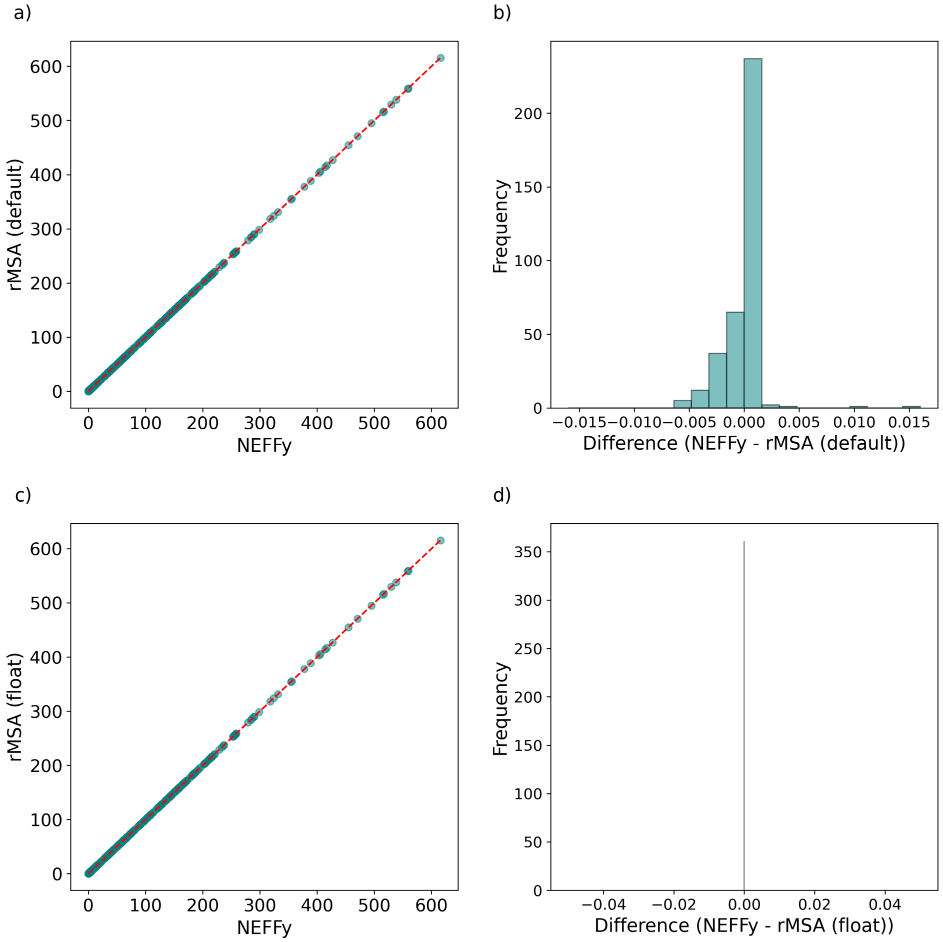

Figure S6: Comparison of NEFF values between `rMSA`, using `double` (default version) and `float` data types, and `NEFFy` (using parameters corresponding to `rMSA`) for the `rMSA` dataset: (**a**, **c**) Scatter plots illustrating NEFF values for `double` (**a**) and `float` (**c**) configurations, with the dotted red line representing the identity line (x = y), indicating equal values for both methods.(**b**, **d**) Histograms showing the distribution of differences for `double` (**b**) and `float` (**d**) configurations.

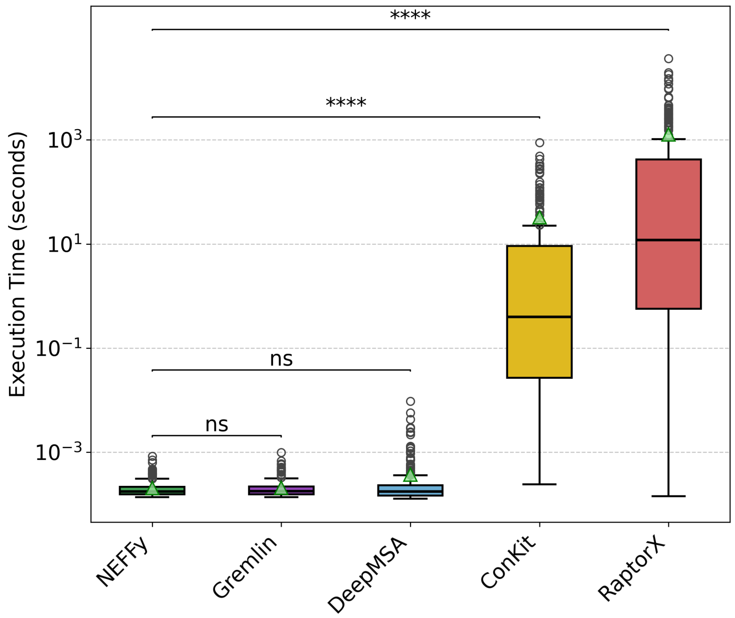

Figure S7: Comparison of execution times (in seconds) for the tools `NEFFy`, `DeepMSA`, `Gremlin`, `Conkit`, and `RaptorX` on a log scale, based on the average from five runs. The mean execution times for each tool are indicated by green triangles. Statistical significance was calculated using one-sided Wilcoxon test, with non-significant differences denoted as "ns" and significant differences represented by asterisks (****).

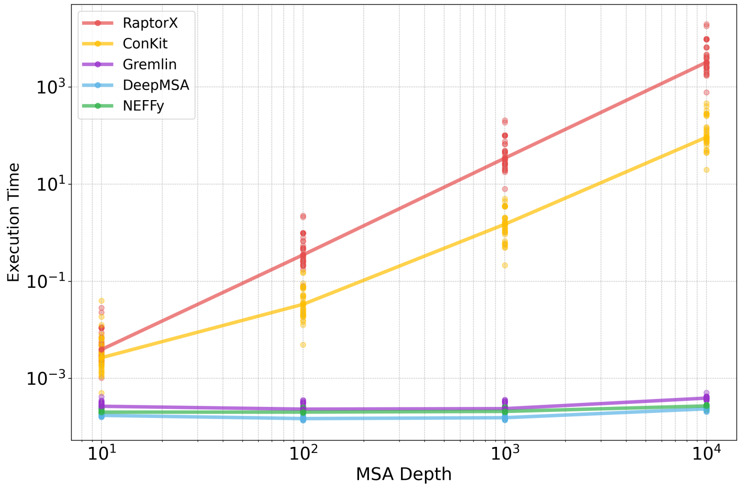

Figure S8: Scalability analysis of five tools—`NEFFy`, `DeepMSA`, `Gremlin`, `Conkit`, and `RaptorX`—based on execution time versus MSA depth for 36 MSA files, each corresponding to a target from CASP15. The plot demonstrates how each tool's performance changes with increasing MSA depth. Execution times were measured across depths from 10 to 10,000, with individual points representing the average execution time (from five runs) per file at each depth. Solid lines show the median execution time across files for each tool.

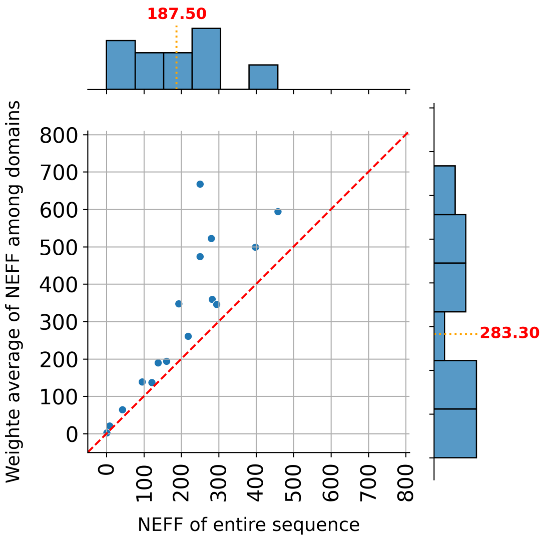

(a)

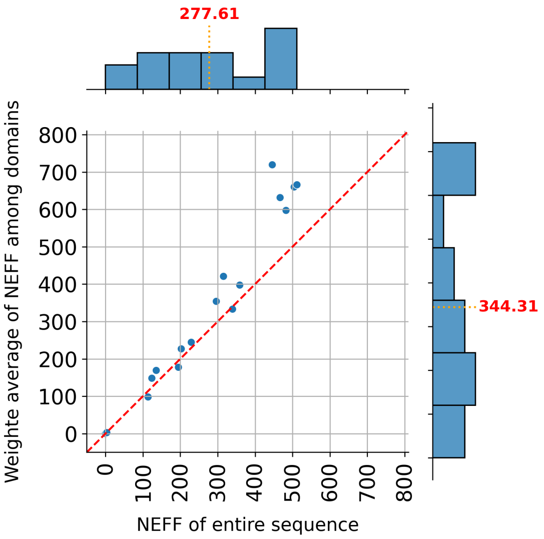

(b)

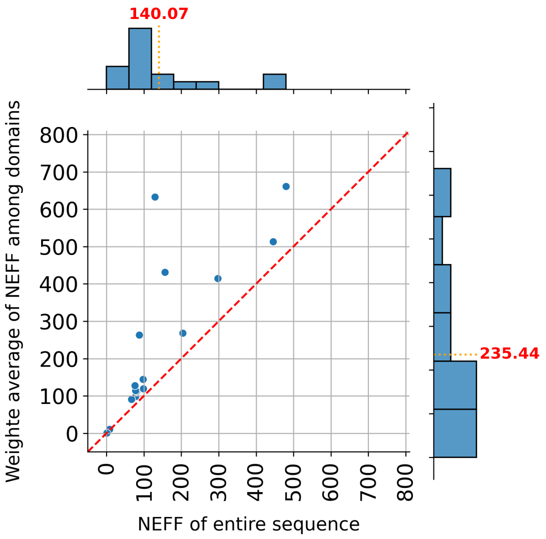

(c)

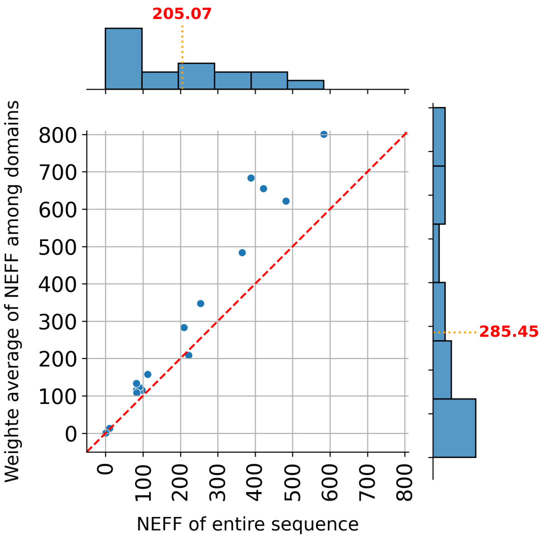

(d)

Figure S9: Domain-level NEFF vs. whole target for 17 multi-domain targets in CASP15 (using normalized NEFF values by **sqrt(L)**), with dotted red line representing the identity line (x = y): **a, b)** AlphaFold: The default pipeline for MSA generation was used, combining three distinct MSAs per target to produce the final MSA. **(a)** Symmetric NEFF **(b)** Asymmetric NEFF **c, d)** RoseTTAFold: The default MSA generation pipeline was employed. **(c)** Symmetric NEFF **(d)** Asymmetric NEFF.

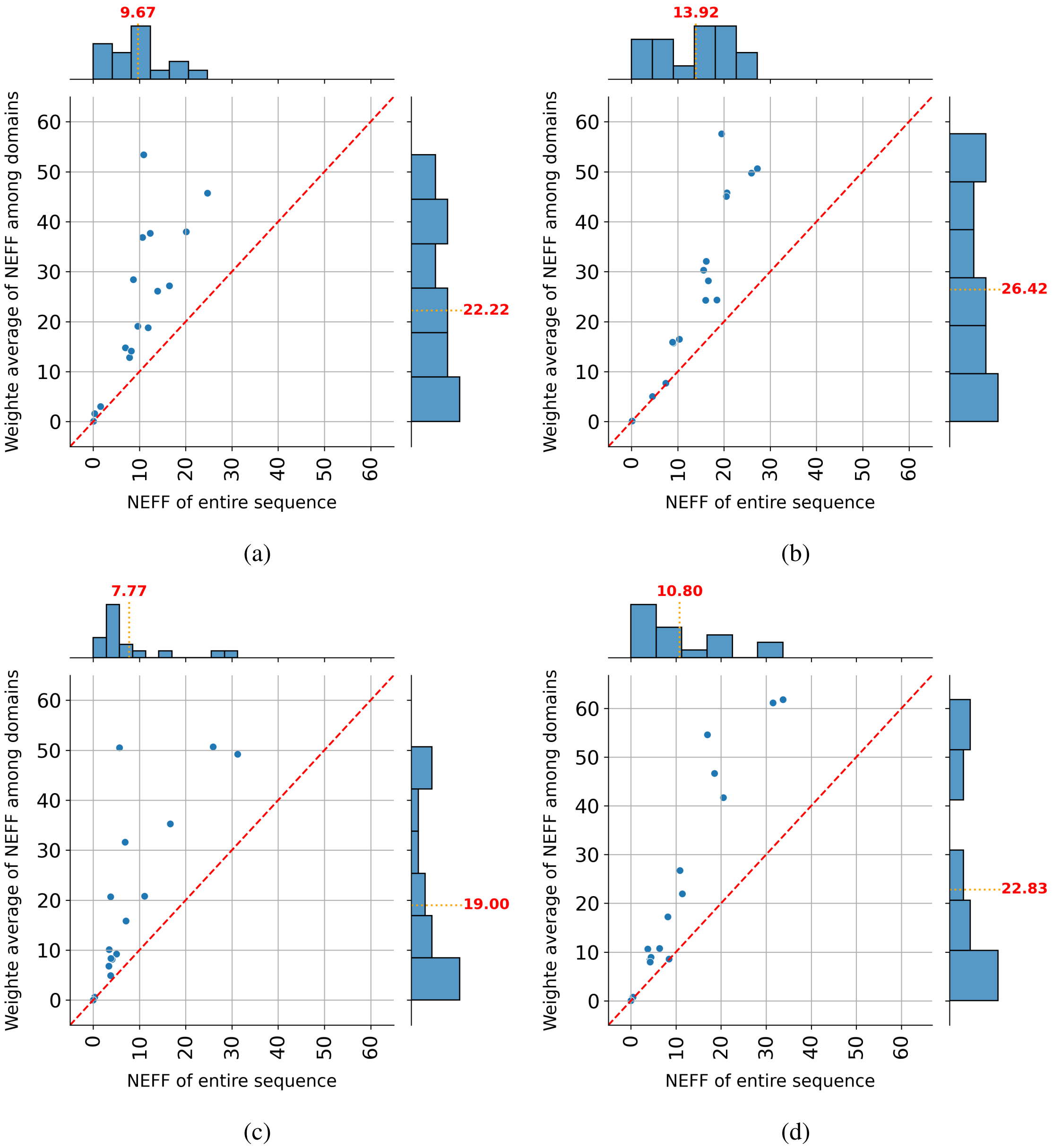

Figure S10: Domain-level NEFF vs. whole target for 17 multi-domain targets in CASP15 (using normalized NEFF values by **L**), with dotted red line representing the identity line (x = y): **a, b)** AlphaFold: The default pipeline for MSA generation was used, combining three distinct MSAs per target to produce the final MSA. **(a)** Symmetric NEFF **(b)** Asymmetric NEFF **c, d)** RoseTTAFold: The default MSA generation pipeline was employed. **(c)** Symmetric NEFF **(d)** Asymmetric NEFF.

depth and execution time. NEFFy, DeepMSA, and Gremlin maintain consistent performance across all tested depths, indicating that these tools are well-optimized and highly scalable for both shallow and deep MSAs, making them particularly suited for large-scale analyses where computational efficiency is critical. In contrast, Conkit and RaptorX exhibit a noticeable increase in execution time with growing depth, following an exponential pattern, which suggests these tools may struggle to scale effectively with deeper MSAs, rendering them less practical for extensive MSA applications compared to the other tools.

S4.4 Case Study on Multi-domain Proteins

Following our analysis of the 19 multidomain sets from the CASP15 dataset, we utilized the target MSAs generated by AlphaFold and RoseTTAFold [Baek et al., 2021], as provided in the paper by Moussad et al. at https://doi.org/10.5281/zenodo.7682977. Since RoseTTAFold generates a single MSA file, we were able to use the provided MSAs directly without any additional steps. For AlphaFold, we used the default AlphaFold 2.3 pipeline, which merges the three MSAs before feeding them into the deep learning model. The combined MSA was subsequently used for our analysis. To obtain domain-level MSAs, we extracted the sequence alignments specific to each domain from the full-length MSA of the target, as detailed in [Wu et al., 2019]. We then used NEFFy to calculate normalized NEFF values for both the full protein and domain-level MSAs, using a similarity threshold θ of 0.8. To getting the NEFF values for individual domains, we utilized the start_pos and end_pos features of NEFFy, setting the domain's start and end positions accordingly. For two of the targets, "T1157s1" and "T1158", NEFF values could not be obtained because one of their domains was fragmented across multiple locations. Therefore, we proceeded with our analysis on 17 such targets.

Figures S9 and S10 display Grishin plots [Kinch et al., 2011] that compare the weighted average NEFF values for MSAs derived from individual domains with the NEFF values for the complete protein chain MSAs, using normalization by sqrt(L) and L, respectively. These plots illustrate the correlation between the weighted sum of the NEFF values for the domains (on the y-axis) and the NEFF values for the entire protein chain (on the x-axis), indicating that the NEFF values of individual domain MSAs are higher than the NEFF values of entire chain MSAs. The observed pattern aligns with the trends noted in prediction accuracy [Moussad et al., 2023].

S5 Alternative NEFF Formulations

S5.1 Clustering-Based NEFF calculation

An alternative method for calculating the effective number of sequences (NEFF) in a multiple sequence alignment (MSA) is based on clustering. In this approach, the number of clusters provides an approximation of the NEFF value. Specifically, each sequence i is assigned a weight

$$w_i = \frac{1}{n_i},$$

where n_i is the number of sequences in the cluster containing sequence i. NEFF is calculated by summing the weights assigned to each sequence:

$$\text{NEFF} = \sum_{i=1}^{N} w_i,$$

where N is the total number of all sequences. Although this procedure is conceptually similar to our NEFF computation, it requires an additional clustering step.

CD-HIT [Fu et al., 2012, Li and Godzik, 2006, Li et al., 2001, 2002] is a widely used tool for obtaining such clusters and has been applied in several studies for NEFF calculation [Bryant et al., 2022, Jones et al., 2015, Kandathil et al., 2019, Kosciolek and Jones, 2016, Yin et al., 2022]. Notably, as described in [Li et al., 2001], CD-HIT clusters unaligned sequences, assuming the availability of a sequence collection rather than relying on pairwise alignments.

Other studies [Jones et al., 2012, Skwark et al., 2014] have used a different clustering method for calculating the effective number of sequences. In this alternative approach, pairwise sequence identities are first computed, and the average identity across all sequence pairs, denoted as $MeanID$, is determined. A similarity threshold ID_{th} is then calculated using the formula:

$$ID_{th} = \min\left(0.5, \frac{0.38 \times 0.32}{MeanID}\right).$$

This threshold is used to decide whether two sequences should be considered redundant; sequences that differ by less than $ID_{th} \times$ length are grouped into the same cluster. As before, each sequence i is given a weight of $w_i = \frac{1}{n_i}$, where n_i represents the number of similar sequences within its cluster, and the overall NEFF is obtained by summing these weights over all sequences.

S5.2 Entropy-Based NEFF Calculation

Several studies compute the number of effective sequences as the exponential of the average entropy calculated over all columns of a multiple sequence alignment (MSA) [Casbon and Saqi, 2004, Peng, 2013, Peng and Xu, 2010, Savojardo et al., 2013]. In this formulation, NEFF can be interpreted as the entropy of the sequence profile derived from the MSA. For proteins, the resulting NEFF is a real value ranging from 1 to 20. An MSA composed of highly similar sequences (or singletons) will have a NEFF value close to 1. At the other extreme, if the amino acids in each column are distributed uniformly, the NEFF value will be close to 20, indicating maximal sequence diversity. More formally, NEFF is defined as:

$$\text{NEFF}(M) = e^{\frac{1}{L} \cdot \text{Entropy}(M)},$$

with

$$\text{Entropy}(M) = -\sum_{i=1}^{L} \sum_{r} p_r^i \ln(p_r^i).$$

where M denotes the MSA of length L, and p_r^i represents the frequency of residue r in column i of the alignment. The summation over r is over all possible residues. Specifically, for protein sequences, r belongs to the set of 20 standard amino acids.

An alternative version of this formulation defines entropy using base-2 logarithms and consequently employs an exponentiation with base 2. Tools such as MMSeq2 [Steinegger and Söding, 2017] and HH-suite [Steinegger et al., 2019] implement NEFF in this manner, and some recent works [Hanson et al., 2019, Lee et al., 2023] have adopted this variation for their NEFF computation.

References

M. Baek, F. DiMaio, I. Anishchenko, J. Dauparas, S. Ovchinnikov, G.R. Lee, J. Wang, Q. Cong, L.N. Kinch, R.D. Schaeffer, et al. Accurate prediction of protein structures and interactions using a three-track neural network. *Science*, 373(6557):871–876, 2021.

M. Baek, R. McHugh, I. Anishchenko, D. Baker, and F. DiMaio. Accurate prediction of nucleic acid and protein-nucleic acid complexes using RoseTTAFoldNA. *bioRxiv*, pages 2022–09, 2022.

P. Bryant, G. Pozzati, and A. Elofsson. Improved prediction of protein-protein interactions using AlphaFold2. *Nat Commun*, 13(1), 2022. doi: 10.1038/s41467-022-29480-5.

J.A. Casbon and M.A. Saqi. Analysis of superfamily specific profile-profile recognition accuracy. *BMC bioinformatics*, 5:1–9, 2004.

R. Evans, M. O'Neill, A. Pritzel, N. Antropova, A. Senior, T. Green, A. Žídek, R. Bates, S. Blackwell, J. Yim, et al. Protein complex prediction with AlphaFold-Multimer. *biorxiv*, pages 2021–10, 2021.

C. Feng, W. Wang, R. Han, Z. Wang, L. Ye, Z. Du, H. Wei, F. Zhang, Z. Prng, and J. Yang. Accurate de novo prediction of RNA 3D structure with transformer network. *bioRxiv*, pages 2022–10, 2022.

L. Fu, B. Niu, Z. Zhu, S. Wu, and W. Li. CD-HIT: accelerated for clustering the next-generation sequencing data. *Bioinformatics*, 2012.

J. Hanson, K. Paliwal, et al. Improving prediction of protein secondary structure, backbone angles, solvent accessibility and contact numbers by using predicted contact maps and an ensemble of recurrent and residual convolutional neural networks. *Bioinformatics*, 35(14):2403–2410, 2019.

D.T. Jones, D.W. Buchan, D. Cozzetto, and M. Pontil. PSICOV: precise structural contact prediction using sparse inverse covariance estimation on large multiple sequence alignments. *Bioinformatics*, 28(2):184–190, 2012.

D.T. Jones, T. Singh, T. Kosciolek, and S. Tetchner. MetaPSICOV: combining coevolution methods for accurate prediction of contacts and long range hydrogen bonding in proteins. *Bioinformatics*, 31(7):999–1006, 2015.

J. Jumper, R. Evans, A. Pritzel, T. Green, M. Figurnov, O. Ronneberger, K. Tunyasuvunakool, R. Bates, A. Žídek, A. Potapenko, et al. Highly accurate protein structure prediction with AlphaFold. *Nature*, 596(7873):583–589, 2021.

M. Källberg, H. Wang, S. Wang, J. Peng, Z. Wang, H. Lu, and J. Xu. Template-based protein structure modeling using the RaptorX web server. *Nature Protocols*, 7(8):1511–1522, 2012.

H. Kamisetty, S. Ovchinnikov, and D. Baker. Assessing the utility of coevolution-based residue–residue contact predictions in a sequence-and structure-rich era. *Proceedings of the National Academy of Sciences*, 110(39):15674–15679, 2013.

SM Kandathil, JG Greener, and D.T. Jones. Prediction of interresidue contacts with DeepMetaPSICOV in CASP13. *Proteins*, 2019.

L.N. Kinch, S. Shi, H. Cheng, Q. Cong, J. Pei, V. Mariani, T. Schwede, and N.V. Grishin. CASP9 target classification. *Proteins*, 79 Suppl 10(Suppl 10):21–36, 2011.

T. Kosciolek and D.T. Jones. Accurate contact predictions using covariation techniques and machine learning. *Proteins*, 2016. doi: 10.1002/prot.24863.

S. Lee, G. Kim, et al. Petascale homology search for structure prediction. *bioRxiv*, 2023.

W. Li and A. Godzik. Cd-hit: a fast program for clustering and comparing large sets of protein or nucleotide sequences. *Bioinformatics*, 22(13):1658–1659, 2006. doi: 10.1093/bioinformatics/btl158. URL `https://doi.org/10.1093/bioinformatics/btl158`.

W. Li, L. Jaroszewski, and A. Godzik. Clustering of highly homologous sequences to reduce the size of large protein databases . *Bioinformatics*, 17(3):282–283, 2001. ISSN 1367-4803. doi: 10.1093/bioinformatics/17.3.282. URL `https://doi.org/10.1093/bioinformatics/17.3.282`.

W. Li, L. Jaroszewski, and A. Godzik. Sequence clustering strategies improve remote homology recognitions while reducing search times. *Protein Eng*, pages 643–649, 2002. doi: 10.1093/protein/15.8.643.

B. Moussad, R. Roche, and D. Bhattacharya. The transformative power of transformers in protein structure prediction. *PNAS*, 120(32):e2303499120, 2023.

R. Pearce, G.S. Omenn, and Y. Zhang. De novo RNA tertiary structure prediction at atomic resolution using geometric potentials from deep learning. *bioRxiv*, pages 2022–05, 2022.

J. Peng. Statistical inference for template-based protein structure prediction. *arXiv preprint arXiv:1306.4420*, 2013.

J. Peng and J. Xu. Low-homology protein threading. *Bioinformatics*, 26(12):i294–i300, 2010.

RCSB Protein Data Bank. Symmetry resources in the PDB. URL `https://www.rcsb.org/docs/general-help/symmetry-resources-in-the-pdb`.

C. Savojardo, P. Fariselli, P.L. Martelli, and R Casadio. Prediction of disulfide connectivity in proteins with machine-learning methods and correlated mutations. *BMC bioinformatics*, 14:1–8, 2013.

F. Simkovic, J.M. Thomas, and D.J. Rigden. ConKit: a python interface to contact predictions. *Bioinformatics*, 33(14):2209–2211, 2017.

M.J. Skwark, D. Raimondi, M. Michel, and A. Elofsson. Improved contact predictions using the recognition of protein like contact patterns. *PLoS computational biology*, 10(11):e1003889, 2014.

M. Steinegger and J. Söding. MMseqs2 enables sensitive protein sequence searching for the analysis of massive data sets. *Nature biotechnology*, 35(11):1026–1028, 2017.

M. Steinegger, M. Meier, et al. HH-suite3 for fast remote homology detection and deep protein annotation. *BMC bioinformatics*, 20:1–15, 2019.

F. Wilcoxon. Individual comparisons by ranking methods. In *Breakthroughs in statistics: Methodology and distribution*, pages 196–202. Springer, 1992.

T. Wu, J. Hou, B. Adhikari, and J. Cheng. Analysis of several key factors influencing deep learning-based inter-residue contact prediction. *Bioinformatics*, 36(4):1091–1098, 2019.

R. Yin, B.Y. Feng, A. Varshney, and B.G. Pierce. Benchmarking AlphaFold for protein complex modeling reveals accuracy determinants. *Protein Science*, 31(8):e4379, 2022.

C. Zhang, W. Zheng, S.M. Mortuza, Y. Li, and Y. Zhang. DeepMSA: constructing deep multiple sequence alignment to improve contact prediction and fold-recognition for distant-homology proteins. *Bioinformatics*, 36(7):2105–2112, 2020.

C. Zhang, Y. Zhang, and AM. Pyle. rMSA: A Sequence Search and Alignment Algorithm to Improve RNA Structure Modeling. *Journal of Molecular Biology*, 435(14):167904, 2023.
